# Divergent Histopathological Networks of Frontotemporal Degeneration Proteinopathy Subytpes

**DOI:** 10.1523/JNEUROSCI.2061-21.2022

**Published:** 2022-05-04

**Authors:** Min Chen, Daniel T. Ohm, Jeffrey S. Phillips, Corey T. McMillan, Noah Capp, Claire Peterson, Emily Xie, David A. Wolk, John Q. Trojanowski, Edward B. Lee, James Gee, Murray Grossman, David J. Irwin

**Affiliations:** ^1^Penn Image Computing and Science Lab, Department of Radiology, Perelman School of Medicine, University of Pennsylvania, Philadelphia, Pennsylvania 19104; ^2^Digital Neuropathology Laboratory, Department of Neurology, Perelman School of Medicine, University of Pennsylvania, Philadelphia, Pennsylvania 19104; ^3^Penn Frontotemporal Degeneration Center, Department of Neurology, Perelman School of Medicine, University of Pennsylvania, Philadelphia, Pennsylvania 19104; ^4^Alzheimer's Disease Research Center, Department of Neurology, Perelman School of Medicine, University of Pennsylvania, Philadelphia, Pennsylvania 19104; ^5^Center for Neurodegenerative Disease Research, Department of Pathology and Laboratory Medicine, Perelman School of Medicine, University of Pennsylvania, Philadelphia, Pennsylvania 19104; ^6^Translational Neuropathology Research Laboratory, Department of Pathology and Laboratory Medicine, Perelman School of Medicine, University of Pennsylvania, Philadelphia, Pennsylvania 19104

**Keywords:** frontotemporal lobar degeneration, histopathology, mediation analysis, network science, tauopathy, TDP-43 proteinopathy

## Abstract

Network analyses inform complex systems such as human brain connectivity, but this approach is seldom applied to gold-standard histopathology. Here, we use two complimentary computational approaches to model microscopic progression of the main subtypes of tauopathy versus TDP-43 proteinopathy in the human brain. Digital histopathology measures were obtained in up to 13 gray matter (GM) and adjacent white matter (WM) cortical brain regions sampled from 53 tauopathy and 66 TDP-43 proteinopathy autopsy patients. First, we constructed a weighted non-directed graph for each group, where nodes are defined as GM and WM regions sampled and edges in the graph are weighted using the group-level Pearson's correlation coefficient for each pairwise node comparison. Additionally, we performed mediation analyses to test mediation effects of WM pathology between anterior frontotemporal and posterior parietal GM nodes. We find greater correlation (i.e., edges) between GM and WM node pairs in tauopathies compared with TDP-43 proteinopathies. Moreover, WM pathology strongly correlated with a graph metric of pathology spread (i.e., node-strength) in tauopathies (*r* = 0.60, *p* < 0.03) but not in TDP-43 proteinopathies (*r* = 0.03, *p* = 0.9). Finally, we found mediation effects for WM pathology on the association between anterior and posterior GM pathology in FTLD-Tau but not in FTLD-TDP. These data suggest distinct tau and TDP-43 proteinopathies may have divergent patterns of cellular propagation in GM and WM. More specifically, axonal spread may be more influential in FTLD-Tau progression. Network analyses of digital histopathological measurements can inform models of disease progression of cellular degeneration in the human brain.

**SIGNIFICANCE STATEMENT** In this study, we uniquely perform two complimentary computational approaches to model and contrast microscopic disease progression between common frontotemporal lobar degeneration (FTLD) proteinopathy subtypes with similar clinical syndromes during life. Our models suggest white matter (WM) pathology influences cortical spread of disease in tauopathies that is less evident in TDP-43 proteinopathies. These data support the hypothesis that there are neuropathologic signatures of cellular degeneration within neurocognitive networks for specific protienopathies. These distinctive patterns of cellular pathology can guide future efforts to develop tissue-sensitive imaging and biological markers with diagnostic and prognostic utility for FTLD. Moreover, our novel computational approach can be used in future work to model various neurodegenerative disorders with mixed proteinopathy within the human brain connectome.

## Introduction

Graph theoretical analyses provide a reliable method using statistical measures (i.e., “network features”) to quantify the topology of brain connections (i.e., “edges”) between regions (i.e., “nodes”) that are useful to study structure, function and model disease in the human brain connectome (Bullmore and Sporns, 2009; Stam, 2014). These approaches are commonly applied to whole brain *in vivo* structural or functional imaging data in neurodegenerative disease research to uncover complex relationships between human brain connectivity and cortical atrophy. While important, this approach is somewhat limited as the gold-standard for diagnosis in neurodegenerative disease is autopsy. Moreover, *in vivo* imaging measures of neurodegeneration are on a macroscopic scale that may not fully reflect the complex cellular processes found in histopathological sampling. Thus, novel analytical tools and approaches are needed to apply network analyses to gold-standard histopathology data directly. This is particularly of importance in frontotemporal lobar degeneration (FTLD) spectrum disorders, which are a common cause of young-onset dementia, and there is limited autopsy data to model disease progression (Irwin et al., 2015).

FTLD is classified neuropathologically into two main proteinopathies that include tauopathies (FTLD-Tau) and TDP-43 proteinopathies (FTLD-TDP; Mackenzie et al., 2010, 2011). While there are group-level associations of specific proteinopathies with some frontotemporal dementia (FTD) clinical syndromes, there is considerable clinical overlap and often difficulty implementing clinical criteria to predict pathology (Murley et al., 2020). Moreover, there is no current neuroimaging or biofluid biomarker able to detect FTLD-Tau or FTLD-TDP pathology *in vivo*. Thus, accurate antemortem diagnosis on an individual patient level is currently not possible, posing a significant obstacle to the implementation of clinical trials for disease-modifying therapies. Detailed postmortem study of human brain histopathology provides an important foundation for biomarker development but these data are traditionally studied using subjective ordinal scales, limiting the ability to perform more advanced statistical modeling.

Here, we address these limitations using novel application of two complimentary computational methods to model microscopic patterns of pathologic accumulation across regions of the human brain using validated digital histopathological measurements (Irwin et al., 2016a; Giannini et al., 2019b) in our large-scale FTLD autopsy cohort. Moreover, we previously found prominent accumulation of tau pathology in juxtacortical white matter (WM) in FTLD-Tau that is relatively distinct from FTLD-TDP across clinical syndromes and proteinopathy subtypes (McMillan et al., 2012; Irwin et al., 2018; Giannini et al., 2021). Therefore, we hypothesize this prominent WM pathology in FTLD-Tau is influential in the cortical spread of pathology in a manner distinct from FTLD-TDP.

First, we apply graph theoretical analyses to our digital pathology data, where nodes are defined as regions sampled and the edges are defined by the correlation strength of the pathologic burden between each possible node pair. This approach is analogous to structural covariance approaches to *in vivo* MRI, where brain regions highly correlated in thickness and morphologic features are inferred to be highly connected (Alexander-Bloch et al., 2013), and here we infer pathologic spread by the strength of an edge (i.e., correlation of pathologic burden) between nodes. Next, we perform mediation analyses, which test zero-order causal-chain relationships between a predictor variable (X), a dependent variable (Y), and the mediation of the X-Y relationship by a third variable (M; Baron and Kenny, 1986). In this analysis, we define X as gray matter (GM) nodes in frontotemporal areas implicated to accumulate pathology relatively early in the disease and Y as a GM node in the posterior parietal cortex thought to accumulate pathology at a later stage of disease in FTLD (Brettschneider et al., 2014; Irwin et al., 2016b). By testing mediation effects of WM pathology averaged between these anterior and posterior regions to approximate long-range tracts, we can model the relative spread of pathology across the brain, where mediation by WM implies greater spread of pathology via long-range WM tracts. Our data suggest tauopathies may propagate via WM axons and glia in a manner distinct from FTLD-TDP.

## Materials and Methods

### Patients

Patients were evaluated clinically at the Penn Frontotemporal Degeneration Center (FTDC) or Penn Alzheimer's Disease Research Center (ADRC) with prospective clinical criteria consensus review by experienced cognitive neurologists and followed in ongoing clinical research programs to autopsy at the Penn Center for Neurodegenerative disease research using standardized methods (Toledo et al., 2014) and criteria (Mackenzie et al., 2010, 2011; Montine et al., 2012) for neuropathological diagnosis.

Patients selected for study all had an FTD dementia syndrome [i.e., behavioral variant FTD (bvFTD), primary progressive aphasia (PPA), amyotrophic lateral sclerosis with FTD (ALS-FTD), corticobasal syndrome (CBS), or progressive supranuclear palsy syndrome (PSPS)] and autopsy confirmation of a primary neuropathological diagnosis of either the most common subtypes of FTLD-Tau [Pick's disease (PiD), corticobasal degeneration (CBD), PSP, tauopathy unclassifiable (Tau-U)] or most common subtypes of FTLD-TDP (subtypes A, B, C, or E). All patients were genotyped for hereditary FTLD based on genetic risk from structured pedigree analysis using repeat-primed PCR for *C9orf72* primed PCR and a multiplexed targeted exome sequencing panel including FTLD-associated genes as described (McCarty Wood et al., 2013). Using modern neuropathologic criteria (Montine et al., 2012), patients with high-level Alzheimer's disease (AD) neuropathologic change (ADNC; i.e., Braak stage B3 with amyloid A3 and C2/3) or cerebrovascular disease co-pathology (i.e., gross infarcts or ≥2 microscopic infarct) were excluded. Our final cohort included 53 FTLD-Tau and 66 FTLD-TDP. Individual patient data are listed in Table 1.

**Table 1.** Patient demographics

	FTLD-Tau	FTLD-TDP
*N* (%female)	53 (47.2%)	66 (47%)
Pathologic subtype	PiD = 13 PSP = 22 CBD = 13 Tau-U = 5	A = 25 B = 22 C = 14 E = 5
Pathogenic mutations	*MAPT* = 6	*GRN* = 15 *C9orf72* = 19 *TBK* = 2
Clinical syndrome	bvFTD = 24 CBS = 5 PPA = 15 PSPS = 9	bvFTD = 54 CBS = 1 PPA = 11
Brain weight (g)	1130.9 (144.3)	1109.1 (186.0)
Postmortem interval (h)	11.3 (7.3)	12.7 (6.5)
Hemisphere sampled	Left = 35 Right = 18	Left = 35 Right = 31
Age at autopsy	68.2 (11.0)	66.0 (9.8)
Disease duration (years)	7.9 (4.1)	6.7 (3.9)
ADNC stage	No ADNC = 33 • A0B0C0 = 10 • A0B1C0 = 12 • A0B2C0 = 4 • A1B0C0 = 3 • A1B0C1 = 1 • A2B0C0 = 2 • A3B0C1 = 1 Low level ADNC = 18 • A1B1C0 = 7 • A1B1C1 = 5 • A1B1C2 = 1 • A1B2C0 = 2 • A2B1C1 = 2 • A3B1C3 = 1 Int. level ADNC = 2 • A2B2C1 = 1 • A2B2C2 = 1	No ADNC = 43 • A0B0C0 = 14 • A0B1C0 = 16 • A0B2C0 = 4 • A1B0C0 = 4 • A1B0C1 = 2 • A2B0C0 = 1 • A2B0C1 = 1 • A2B0C2 = 1 Low level ADNC = 19 • A1B1C0 = 9 • A1B1C1 = 3 • A1B2C0 = 1 • A1B2C1 = 1 • A2B1C1 = 1 • A2B1C2 = 2 • A3B1C2 = 1 • A3B1C3 = 1 Int. level ADNC = 4 • A2B2C2 = 2 • A2B3C2 = 1 • A3B2C0 = 1
Copathologies	Hippocampal sclerosis = 1 LBD brainstem = 5 LBD transitional = 1 LBD neocortical = 1 ARTAG = 23 AGD = 1	Hippocampal sclerosis = 6 LBD amygdala = 3 LBD brainstem = 5 LBD transitional = 1 LBD neocortical = 1 ARTAG = 9 AGD = 3

Cells denote frequency of patients per category or mean (SD) for continuous variables. Independent Student's *t* tests or nonparametric Mann–Whitney *U* were used for continuous data and χ^2^ analysis for categorical data.

*N* = number, %F = percentage of patients with female sex, AD stage = stage of Alzheimer's disease neuropathologic change (ANDC) co-pathology according to current neuropathological criteria (A = Thal amyloid phase, B = Braak tau stage, C = Consortium to Establish a Registry for Alzheimer's disease CERAD senile plaque score), Co-pathology = frequency of comorbid neuropathological diagnoses, FTLD-Tau = tauopathies, FTLD-TDP = TDP-43 proteinopathies, PiD = Pick's disease, PSP = progressive supranuclear palsy, CBD = corticobasal degeneration, Tau-U = unclassifiable tauopathy, A–E = FTLD-TDP subtypes, *MAPT* = microtubule-associated protein Tau, *GRN* = progranulin, *C9orf72* = hexanucleotide expansion mutation in C9orf72 gene, *TBK* = *TANK-binding kinase 1*, bvFTD = behavioral variant frontotemporal dementia, CBS = corticobasal syndrome, PPA = primary progressive aphasia, PSPS = PSP syndrome, ARTAG = aging-related Tau astrogliopathy, AGD = argyrophilic grain disease, LBD = Lewy body disease stage.

*MAPT* includes one patient each with p.G389R, p.L266V and p301L and three patients with c.915 + 16C>T.

### Digital histopathology

Paraffin embedded tissue samples analyzed for digital histology were obtained fresh at autopsy and cut into 6-µm sections and stained for phosphorylated-tau (AT8; Thermo Scientific; 1:1000 dilution without antigen retrieval) in FTLD-Tau and phosphorylated-TDP-43 (p409/410; Millipore; 1:1000 dilution with citric acid antigen retrieval at 99° for 20 min) in FTLD-TDP using previously described methods in the Penn Digital Pathology Lab (Irwin et al., 2016a). Brain sampling included core regions in the anterior frontotemporal regions [orbitofrontal cortex (OFC), Brodmann area (BA)11, midfrontal cortex (MFC), BA46, superior temporal cortex (STC), BA22, anterior cingulate gyrus (ACG), BA24] and a more posterior relatively spared region (angular, ANG, BA39) from within a single hemisphere. In more recent autopsies, extended sampling from other cortical regions implicated in FTD was performed (Giannini et al., 2019a) and used in analysis. Any ripped or damaged tissue that precluded digital measurement was excluded. All available data are reported in Table 2. We added entorhinal cortex (BA28) in those patients with available tissue (FTLD-Tau *n* = 47 GM, *n* = 48 WM; FTLD-TDP *n* = 35 GM, *n* = 39 WM) for an exploratory analysis of hippocampal spread because of the unique connectivity of this limbic region and importance of hippocampal pathology on FTD symptoms (Scarioni et al., 2022).

**Table 2.** Regional digital pathologic measurements

	Region	Node type	FTLD-Tau *N* = 53	FTLD-TDP *N* = 66
Anterior ventral frontal	Anterior insula (BA13)	GM	0.96 (1.75) *N* = 11	−2.15 (1.18) *N* = 17
		WM	1.04 (1.84) *N* = 11	−3.71 (1.25) *N* = 18
	Orbitofrontal cortex (BA11)	GM	0.01 (1.28) *N* = 34	−2.54 (1.66) *N* = 60
		WM	−0.37 (1.13) *N* = 34	−4.74 (1.69) *N* = 62
Anterior dorsolateral/medial frontal	Dorsolateral prefrontal cortex (BA9)	GM	1.96 (2.51) *N* = 10	−3.29 (1.50) *N* = 12
		WM	1.19 (2.36) *N* = 11	−4.42 (1.69) *N* = 13
	Inferior frontal cortex (BA44)	GM	2.26 (2.37) *N* = 10	−2.56 (1.23) *N* = 7
		WM	1.83 (2.48) *N* = 10	−3.50 (1.15) *N* = 8
	Inferior prefrontal cortex (BA45)	GM	0.92 (2.13) *N* = 6	−2.76 (1.28) *N* = 13
		WM	0.37 (2.33) *N* = 9	−4.40 (1.70) *N* = 14
	Midfrontal cortex (BA46)	GM	0.94 (2.07) *N* = 42	−2.94 (1.38) *N* = 52
		WM	0.41 (2.94) *N* = 42	−4.58 (1.96) *N* = 56
	Anterior cingulate cortex (BA24)	GM	0.79 (3.03) *N* = 29	−2.41 (1.50) *N* = 54
		WM	−0.11 (3.75) *N* = 31	−4.74 (1.83) *N* = 59
	Medial prefrontal cortex (BA32)	GM	1.48 (3.67) *N* = 7	−2.55 (0.97) *N* = 12
		WM	0.81 (3.73) *N* = 9	−4.43 (2.06) *N* = 13
Anterior-mid temporal	Anterior inferior temporal cortex (BA20)	GM	1.44 (4.83) *N* = 7	−2.98 (1.92) *N* = 17
		WM	0.49 (4.66) *N* = 11	−5.09 (2.05) *N* = 17
	Superior temporal cortex (BA22)	GM	−0.14 (4.55) *N* = 36	−2.90 (1.48) *N* = 54
		WM	−0.55 (4.37) *N* = 36	−4.83 (1.98) *N* = 54
Posterior parietal	Angular gyrus (BA39)	GM	0.67 (5.01) *N* = 33	−3.05 (1.76) *N* = 53
		WM	−0.15 (5.01) *N* = 36	−5.14 (1.79) *N* = 57
	Posterior cingulate cortex (BA23)	GM	0.82 (5.71) *N* = 5	−2.49 (1.72) *N* = 8
		WM	−0.50 (5.44) *N* = 3	−4.96 (1.91) *N* = 8
	Superior parietal cortex (BA5)	GM	0.68 (5.83) *N* = 16	−3.28 (1.64) *N* = 23
		WM	−0.53 (5.44) *N* = 15	−4.69 (1.70) *N* = 22

Cells depict the mean natural log transformed percent area occupied (%AO) digital pathologic measurement of tau (in FTLD-Tau) or TDP-43 (in FTLD-TDP) pathologic inclusions and SD in parenthesis. *N* = number of available tissue measurements, BA = Brodmann area, GM = grey matter, WM = white matter. Note, since %AO measurements are dependent on morphologic features of pathology, we do not perform direct comparisons of %AO data between FTLD-Tau and FTLD-TDP proteinopathy groups.

Whole slide images were acquired in the Penn Digital Pathology Lab on a digital slide scanner (Aperio AT2, Leica Biosystem) at 20× magnification. Images were digitally analyzed using QuPath software (version v0.2.0) to calculate the percentage of area occupied (%AO) of pixels with pathologic tau or TDP-43 in random sampling of representative GM and adjacent relative deep WM as published previously (Giannini et al., 2019a, 2021). To account for staining batch effects, we stained all tissue in close temporal proximity and employed a custom digital image analysis algorithm optimized for each staining run as previously validated (Giannini et al., 2019b).

### Experimental design and statistical analysis

#### General methods

This is a retrospective autopsy cohort study. Demographics were compared between groups using standard univariate statistics for parametric (independent Student's *t* tests) or non-parametric (Mann–Whitney *U*) for continuous data or categorical data (χ^2^) as appropriate. A normal distribution was obtained for %AO pathology data using a natural log transformation. All statistical analyses were performed using R (version 4.0.3) and two-tailed statistics reporting a significance level of *p* < 0.05 based on the hypothesis-driven nature of our analyses.

#### Statistical analyses, graph theoretical analysis

We constructed a weighted non-directed graph for each of the FTLD proteinopathy groups. Nodes in the graph represent the GM or WM regions where histopathology AO% were sampled (Table 2). The edges in the graph are weighted using the group-level Pearson's correlation coefficient between the %AO measurements for each pairwise node comparison. We compared graphs between groups using a Fisher's transformation of the r coefficient into a *z* score to test group-level differences between individual node pair correlations with a z-test (Fisher, 1915). This approach accounts for potential discrepancies in the number of pairwise datapoints available for calculating the edge weights in each group. Since our hypothesis was based on overall patterns of disease between GM and WM nodes and not at specific regional nodes, we used a statistical threshold of <0.05 to denote group-level differences in node pair correlation in the graph.

Correlation matrices do not account for the overall severity of the pathology at a given node (e.g., there can be high correlation between two nodes with low pathology), thus we calculated group-level weighted-degree (i.e., node strength) for each node and tested the correlation of this network feature of node integration with the average %AO pathology measurement at each node for FTLD-Tau and FTLD-TDP. Our histopathology networks include a single measurement per node [i.e., %AO from each GM and adjacent WM region of interest (ROI) per slide image] for each individual, thus we cannot calculate individual patient-level network metrics and instead use averaged metrics for each group for comparisons for this analysis; node strength is defined as the sum of the group-level edge weights at each node. In our network, this is represented by the sum of the Pearson's correlation coefficients from all possible node pair combinations in the graph for a given node. We interpret node strength as the relative importance of a node in pathologic spread among the network, where higher node strength implies greater role in mediating pathologic spread between nodes. This is particularly relevant for nodes with high pathology burden, where strong correlations with adjacent nodes can imply potential spread of pathology.

#### Statistical analyses, mediation analysis

To test the association of WM connectivity on the distribution of GM pathology we investigated a unique application of causal mediation analyses to our postmortem digital pathology data. Mediation analysis uses regression modeling to test the association of a mediator variable (M) on a hypothesized causal interaction between a predictor variable (X) and a dependent variable (Y; Baron and Kenny, 1986). We refer to the original, premediated relationship between X and Y as the total effect (c). M is considered a full mediator for the relationship between X and Y when the total effect is no longer significant post mediation. However, if there is still a significant residual relationship (known as the direct effect, c') between X and Y after mediation, this results in a partial mediation effect. In both cases, we also examine the indirect effect (a*b), which is an evaluation of the relationship between X and Y as a combination of their individual relationship with the mediator (i.e., X -> M and M -> Y). To evaluate the indirect effect, we use a bootstrapping approach (bootstrap sample = 1000, confidence interval = 95%, implemented in R v4.0.3) which has been shown to be more effective for data with limited sample size (Preacher and Hayes, 2004; Hayes, 2009). When the indirect effect is non-significant, then there is an absence of a mediation effect.

We tested the association between %AO of measured pathology in anterior frontotemporal GM nodes as the X variable and the posterior parietal GM node as the Y dependent variable. To approximate the WM tracts that connect these regions as the mediator variable (M) we averaged %AO pathology from corresponding regions of deep WM on each slide image between the X and Y nodes. Mediation effects and were calculated from the regression coefficients in univariate and multivariate regression models to predict GM %AO in posterior parietal lobe (Y variable).

### Code accessibility

Data and codes used in analysis are available from corresponding author.

## Results

### Patient groups and regional pathology data

Patients included for study included 53 FTLD-TDP and 66 FTLD-Tau patients. Patient groups had similar age, disease duration, sex distribution and other demographic features (*p* > 0.05). The most common clinical presentation was bvFTD in both groups and the pathologic and genetic subgroups comprising each proteinopathy class are listed in Table 1.

**Figure 1.**
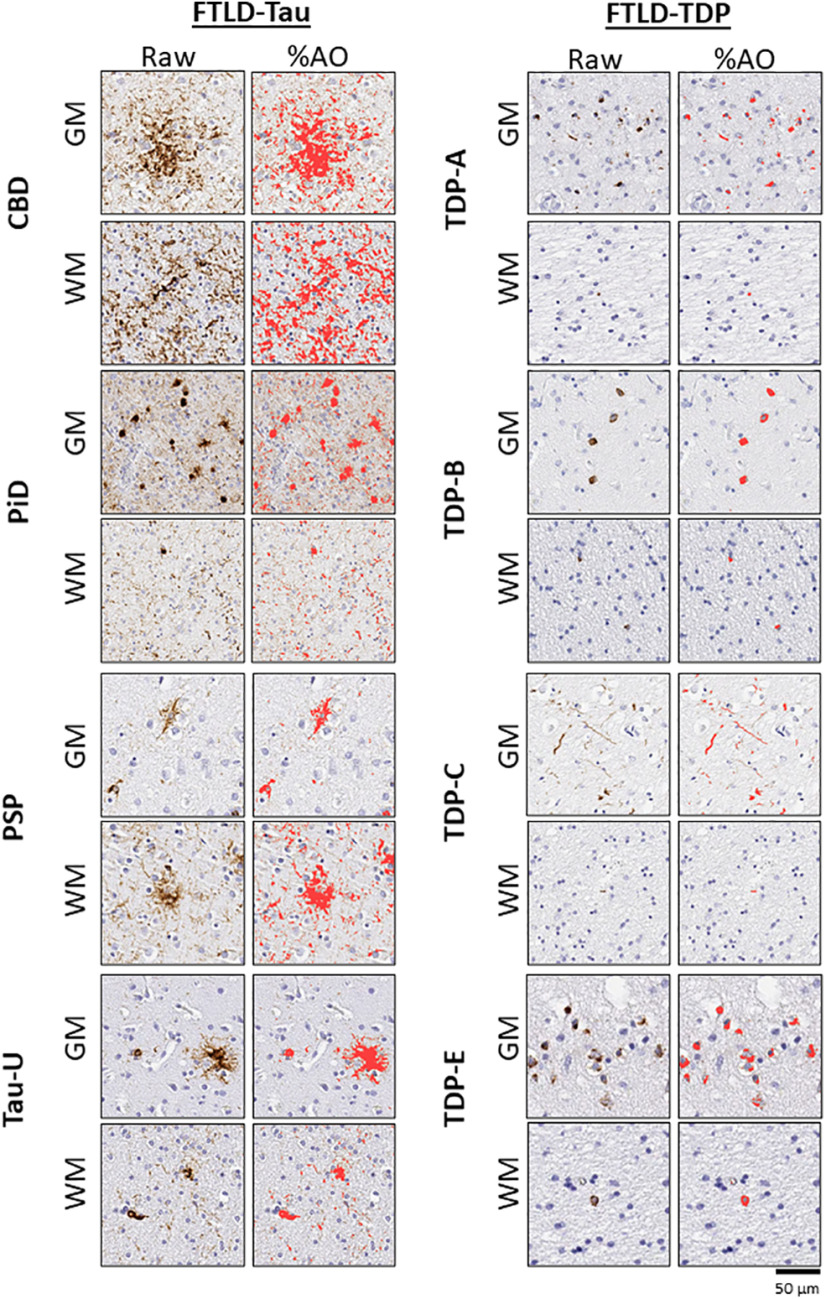
Digital histopathological analysis. Photomicrographs depict representative raw image and %AO of positive pixel digital quantification (%AO, red overlay) from MFC (BA46) grey (GM) and white matter (WM) in FTLD-Tau subtypes (CBD; PiD; PSP; Tau-U) and FTLD-TDP subtypes (TDP A, B, C, E). Scale bar: 50 µm.

First, we examined the relative density of pathology (i.e., %AO) across GM and WM regions in both proteinopathy groups (Table 2). Similar to our previous findings (McMillan et al., 2012; Irwin et al., 2018; Giannini et al., 2021), average WM %AO was relatively equivalent to adjacent GM in FTLD-Tau, whereas FTLD-TDP had greater relative pathology in GM compared with WM (Fig. 1). Areas of highest pathology in FTLD-Tau included dorsolateral and medial frontal neocortical (i.e., BA9, BA44, BA45, BA46, BA32) and limbic frontal (i.e., BA24) GM and WM regions, whereas FTLD-TDP greatest pathology was observed in ventral neocortical (BA11) and limbic (BA13) frontal GM regions as well as anterior temporal neocortical regions (BA20, BA22). There were shared regions of relatively high pathology for both FTLD-Tau and FTLD-TDP in the anterior insula (BA13), ACG (BA24), and some dorsolateral frontal regions. Not surprisingly, more posterior parietal regions had relatively mild pathology in both groups except for limbic posterior cingulate cortex in FTLD-TDP.

### Graph theoretical analyses

**Figure 2.**
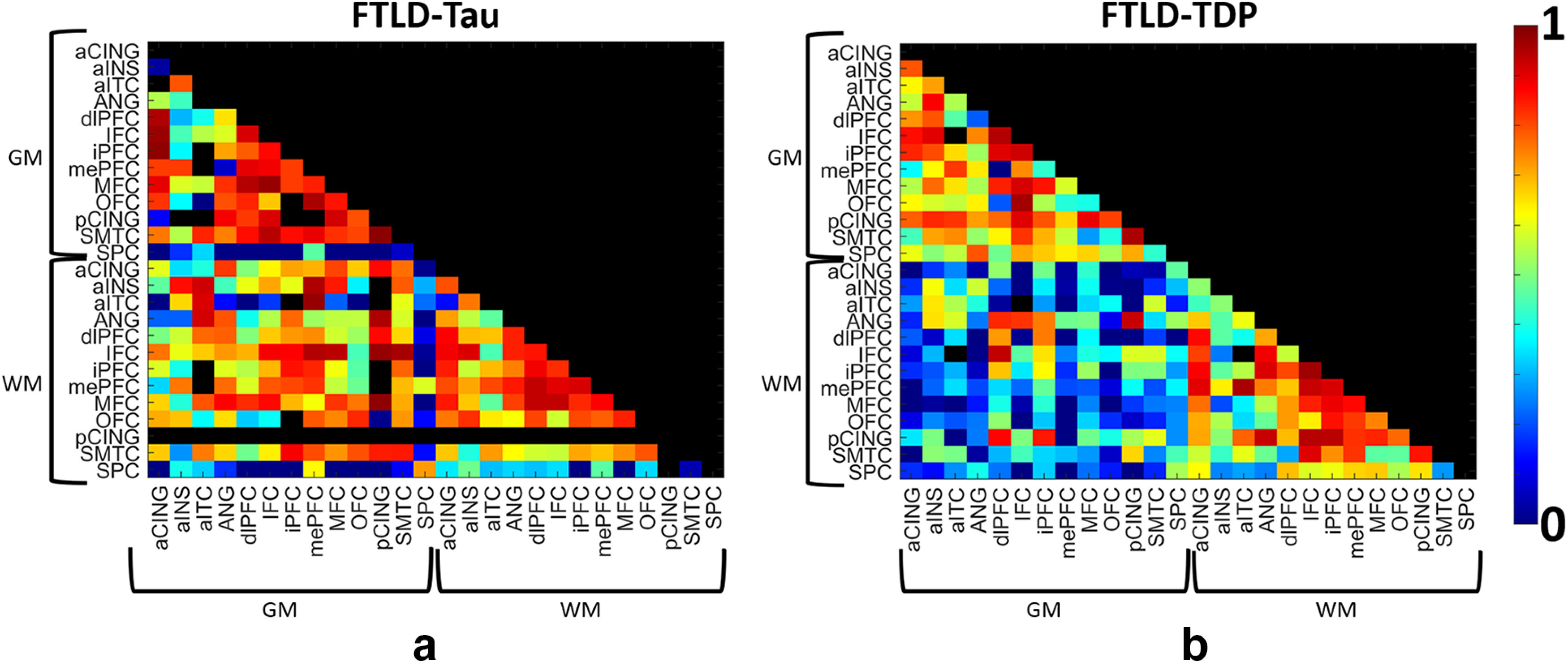
Correlation matrices of digital histopathological measurements across grey matter (GM) and white matter (WM) nodes in FTLD-Tau and FTLD-TDP. Heat map depicts the Pearson's correlation coefficient between nodes according to sidebar scale in (***a***) FTLD-Tau and (***b***) FTLD-TDP proteinopathy groups. We depict the lower half of the map only to avoid redundancy. Node pairs with insufficient sample sizes to evaluate a correlation coefficient are excluded from the analysis and shown as black.

We first performed a correlation matrix to examine associations of pathologic burden between GM and WM node pairs in each group (Fig. 2). Overall, FTLD-Tau pathology was strongly correlated between and within most GM and WM regions while in FTLD-TDP there was strong correlation among GM and among WM nodes but weak correlation between GM and WM node pairs (Fig. 2).

**Figure 3.**
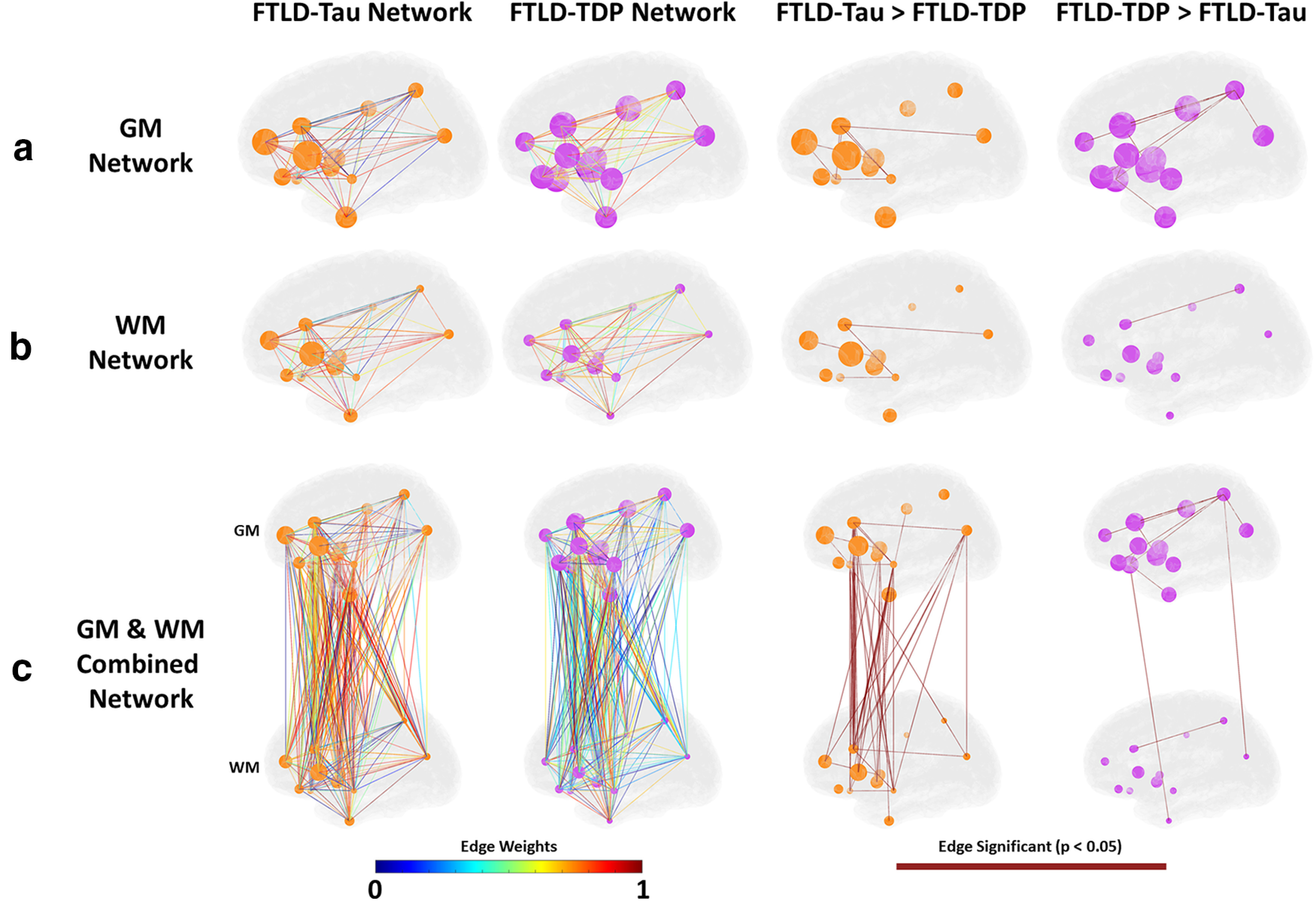
Digital histopathology graphs for FTLD-Tau and FTLD-TDP. Graphs depict (***a***) grey matter (GM), (***b***) white matter (WM), and (***c***) GM+WM nodes in each proteinopathy group. Node size is depicted as total amount of relative pathology measured at each node (larger node = higher percentage of area occupied (%AO) positive pixels of pathology). Edges between nodes are represented by the strength of the Pearson correlation between nodes according to the edge weight heat map. Differences between groups are depicted as edges surviving 0.05 statistical threshold in a one-tailed comparison between the correlations.

**Figure 4.**
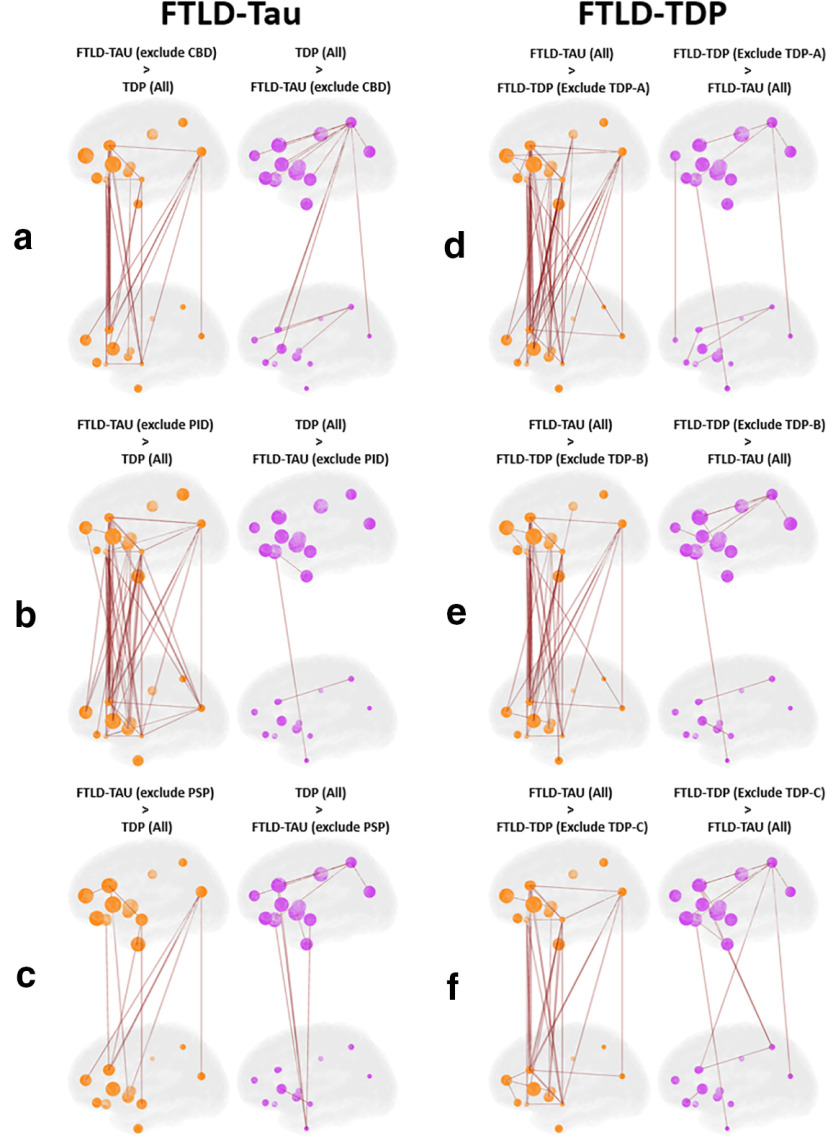
Sensitivity analysis of pathologic subtypes in FTLD-Tau compared with subtypes of FTLD-TDP. Graphs depict GM+WM nodes for cross-validations where we repeated the FTLD-Tau and FTLD-TDP GM+WM histopathology network comparisons while excluding a different subgroup from each analysis: (***a***) FTLD-Tau group excluding CBD subtype versus FTLD-TDP total group; (***b***) FTLD-Tau group excluding PiD subtype versus FTLD-TDP total group; (***c***) FTLD-Tau group excluding PSP subtype versus FTLD-TDP total group; (***d***) FTLD-Tau total group versus FTLD-TDP group excluding TDP subtype A; (***e***) FTLD-Tau total group versus FTLD-TDP group excluding TDP subtype B; (***f***) FTLD-Tau total group versus FTLD-TDP group excluding TDP subtype C. Node size is depicted as total amount of relative pathology measured at each node (larger node = higher percentage of positive pixels of pathology, %AO). Differences between groups are depicted as edges surviving 0.05 statistical threshold in a one-tailed comparison between the correlations.

**Figure 5.**
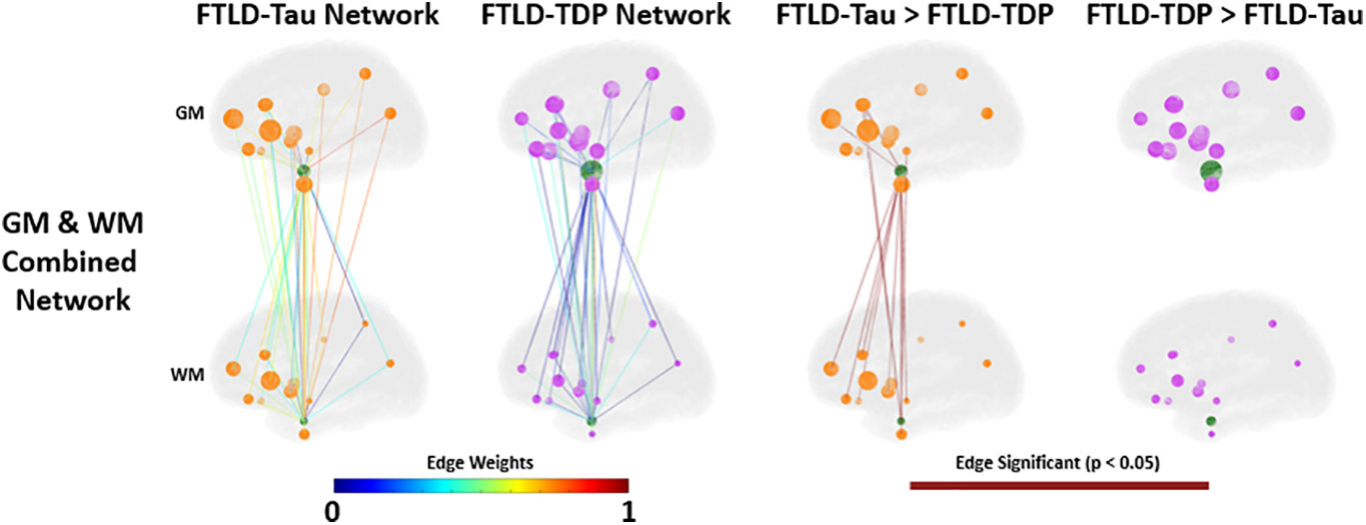
Exploratory analysis of hippocampal connectivity. Graphs depict GM+WM associations of hippocampus entorhinal cortex (BA28; green node) with the cortical regions sampled. Node size is depicted as total amount of relative pathology measured at each node (larger node = higher percentage of positive pixels of pathology, %AO). Edges between nodes are represented by the strength of the Pearson correlation between nodes according to the edge weight scale. Differences between groups are depicted as edges surviving 0.05 statistical threshold in a one-tailed comparison between the correlations.

Next, we constructed a weighted non-directed graph for each proteinopathy group where each region sampled was designated as a node and the correlation coefficient from Figure 1 between each node pair is interpreted as the edge. This approach allows us to visualize microscopic patterns of disease within the macroscopic topology of the human brain (Fig. 3) to compare anatomic distribution of node pair correlation (i.e., hypothesized spread of disease) between groups. Analyses of *in vivo* brain connectivity traditionally use structural measure of WM tracts or functional co-activation as a measure of edges between GM nodes (Rubinov and Sporns, 2010); however, in our analysis here we do not measure brain connectivity directly as this approach is limited in human postmortem brain samples. Instead, we examine the distribution of pathology as a measure of spread throughout the network of GM and WM regions. Therefore, we designate both GM and WM regional %AO data as individual nodes. To examine differential associations of GM and WM pathology to overall patterns in the brain, we first analyzed graphs of GM and WM pathology independently and then examined a combined graph integrating data from both regional GM and WM nodes. Comparison of GM graphs finds overall greater number of significantly stronger edges in FTLD-TDP versus FTLD-Tau, which were most prominent between ventral and dorsolateral frontal regions and posterior parietal lobe, whereas GM associations in FTLD-Tau were strongest among dorsolateral frontal GM nodes with high tau pathology in FTLD-Tau (Fig. 3*A*). WM nodes were similarly interrelated in both groups (Fig. 3*B*), whereas the graphs incorporating both GM and WM nodes had much more robust edges (i.e., associations) between GM and WM node pairs across both anterior and posterior nodes in FTLD-Tau versus FTLD-TDP (Fig. 3*C*). Next, we performed cross-validation sensitivity analyses where we repeated the FTLD-Tau and FTLD-TDP GM+WM histopathology network comparisons while excluding each main proteinopathy subgroup; we found similar results of overall greater correlation of GM-WM node pairs (i.e., greater number of significant GM-WM edges) in each FTLD-Tau versus FTLD-TDP subgroup comparison (Fig. 4). Finally, we performed an exploratory analysis testing disease spread from the entorhinal cortex (BA28) GM to other cortical regions and found similar greater correlation of BA28 GM with WM of other cortical regions in FTLD-Tau compared with FTLD-TDP (Fig. 5).

**Figure 6.**
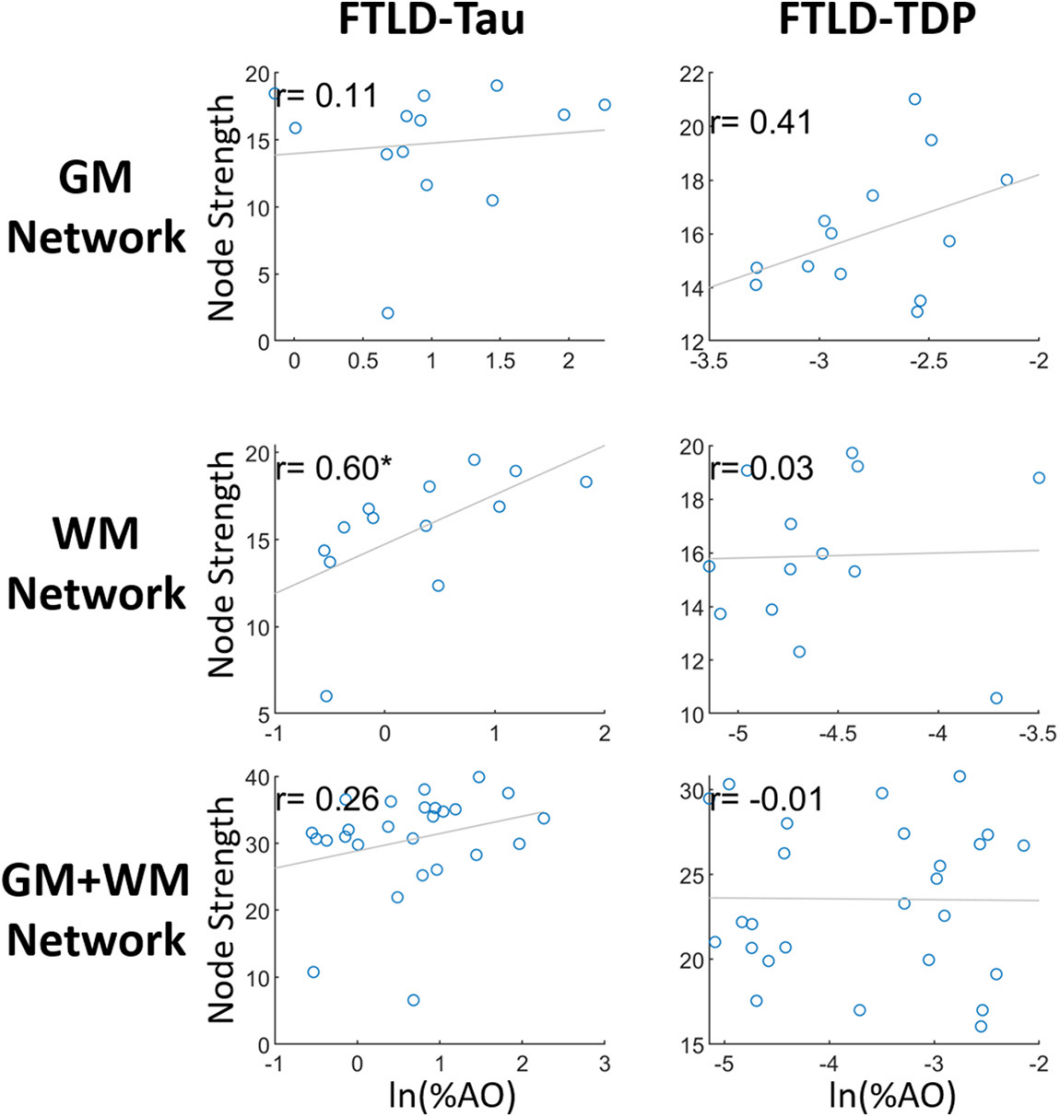
Comparison of node strength with grey matter (GM) and white matter (WM) node pathologic burden in FTLD-Tau and FTLD-TDP. Scatterplots depict group level average digital pathology measurement (i.e. natural log of positive percentage of pixels for pathology, ln(%AO)) plotted by average node strength for GM and WM cortical nodes. Node strength graphically correlates with GM disease burden in FTLD-TDP > FTLD-Tau but does not reach statistical significance, while FTLD-Tau WM pathologic burden has positive correlate with node strength. These data suggest differential contributions of GM and WM pathology to distribution of pathology in the brain for FTLD proteinopathies.

These edges, which are based on the adjacency matrix of Pearson correlations between nodes, do not account for overall disease severity. Therefore, pathologic spread between nodes could be falsely inferred from a high correlation between two nodes with low or negligible pathologic burden. As such, we calculated the graph metric, node strength, as the sum of all edges at each node in the pathology graphs for each proteinopathy group (please see methods) to quantify overall node integration within the graphs and tested their association with GM and WM %AO. We find WM %AO for tau is strongly correlated with node strength in FTLD-Tau (*r* = 0.60, *p* < 0.03), whereas in FTLD-TDP WM %AO was not associated with node strength (*r* = 0.03, *p* = 0.9). In contrast, FTLD-TDP GM %AO had a stronger graphical association with node strength than FTLD-Tau although this did not reach statistical significance (Fig. 6).

### Mediation analyses

**Figure 7.**
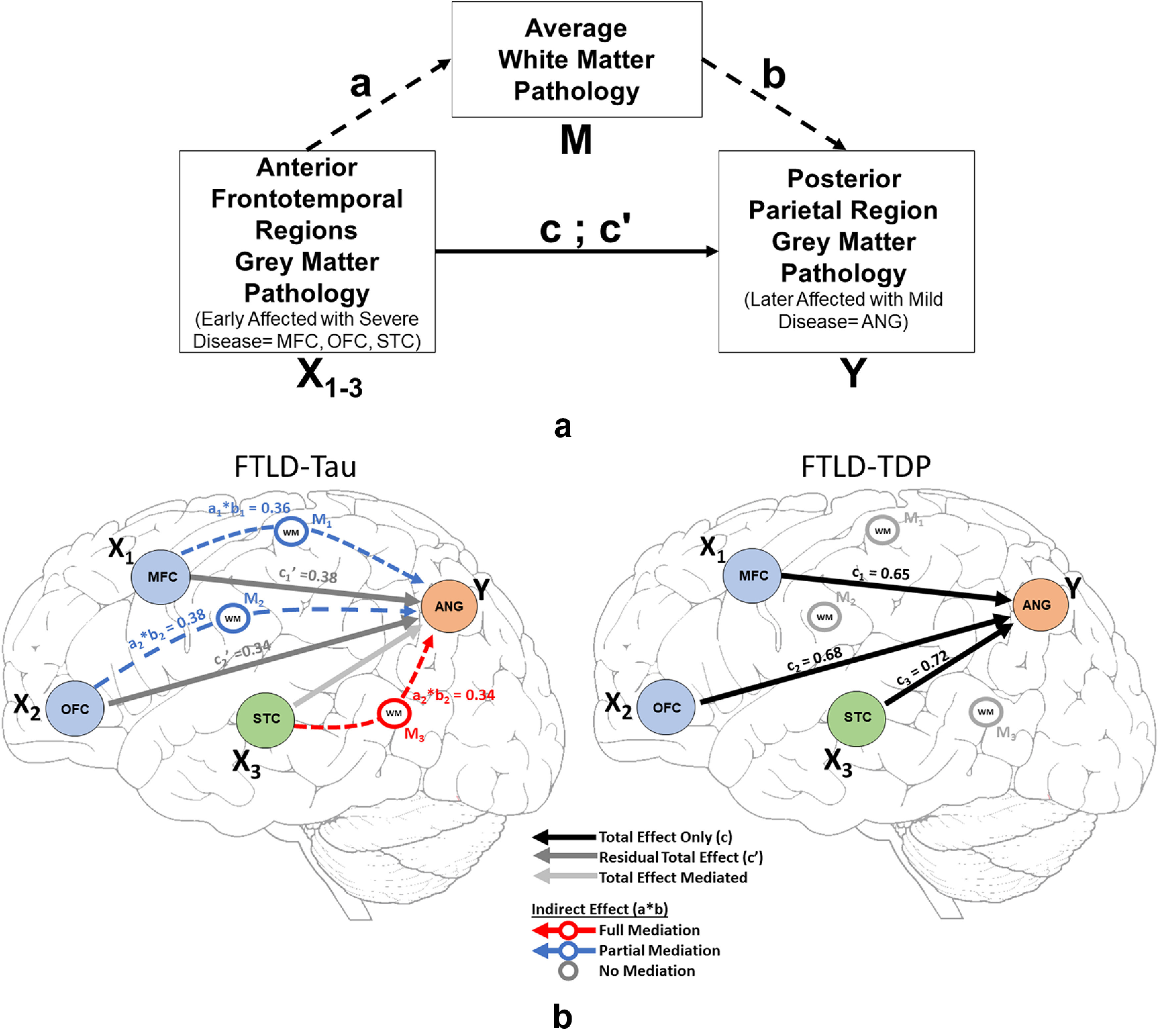
Mediation analysis of WM pathology on associations between anterior frontotemporal and posterior parietal cortex in FTLD-Tau and FTLD-TDP. Schematic in ***a*** depicts the mediation analysis for testing the association of anterior frontotemporal GM nodes (orbitofrontal cortex = OFC, midfrontal cortex = MFC, superior temporal cortex = STC) and posterior parietal grey matter (GM) node (ANG = angular gyrus). The mediation analysis tests the mediation of average white matter (WM) measurement (designated as M) between anterior frontotemporal GM regions (designated as X) compared with distal parietal GM (designated as Y). Diagrams in ***b*** show the mediation effect (or lack of) for each of these tests in FTLD-Tau (left) and FTLD-TDP (right).

Next, we performed a focused mediation analysis to test the specific influence of WM pathology between the density of pathology in anterior frontotemporal GM nodes in our core regions sampled, where disease is likely an earlier event, and the more posterior ANG parietal region, where pathology is likely to accumulate later in disease progression (Brettschneider et al., 2014; Irwin et al., 2016b). In these analyses, we used the average %AO between juxtacortical WM measurements between nodes as an approximation of the WM tracts connecting GM nodes as our mediator variable (M). We found evidence for full or partial mediation by WM pathology on the association of all three anterior frontotemporal regions with ANG GM in FTLD-Tau, while we did not observe mediation effects for WM in FTLD-TDP (Fig. 7). This suggests accumulation of tau pathology in long-range association tracts is influential in the relationship between the accumulation of pathology in anterior frontotemporal regions and more posterior parietal lobe.

## Discussion

Here, we employ complimentary computational approaches of graph theoretical and mediation analyses to model FTLD proteinopathy disease spread in the human brain. This novel approach was facilitated by our validated objective digital measurements of gold-standard histopathology, as traditional univariate approaches with subjective ordinal ratings commonly used in autopsy studies may not capture complex interactions in the human brain that can be more easily interrogated using a network science approach. First, graph theoretic analyses found network feature of node integration (i.e., node-strength) associated more strongly with disease burden in WM for FTLD-Tau but not for FTLD-TDP. Moreover, there was overall greater correlation of GM-WM node pairs in FTLD-Tau compared with FTLD-TDP, suggesting greater influence of WM pathology in spread of tauopathy than TDP-43 proteinopathy. Next, mediation analyses found mediation effects of WM pathology on the association between pathology in anterior and posterior GM nodes in FTLD-Tau, but not in FTLD-TDP. These data suggest divergent microscopic patterns of propagation via WM in tauopathies compared with FTLD-TDP.

Regional measurements of microscopic pathology in our dataset (Table 2) find greater overall relative WM pathologic burden in FTLD-Tau. Moreover, our pathologic measurements reflect known gross patterns of disease in FTLD (Broe et al., 2003) but highlight different general proclivities for microscopic regional pathology between groups, particularly in the frontal lobe where dorsolateral and medial frontal neocortical and limbic regions were more affected in FTLD-Tau compared with more prominent ventral frontal neocortical and limbic regions in FTLD-TDP. These data recapitulate previous findings from our group (Irwin et al., 2018; Giannini et al., 2021) and others (Kim et al., 2020; Mackenzie and Neumann, 2020), highlighting the reproducibility of our digital methods. There was also partial overlap in regional burden between FTLD-Tau and FTLD-TDP, where anterior insula and cingulate gyrus both had relatively severe pathology in both groups. These regions have been implicated in behavioral manifestations of FTD associated with selective loss of specialized neurons for emotional processing (Seeley et al., 2006). Therefore, tau and TDP-43 proteinopathies may converge in specific cellular populations in large-scale neurocognitive networks but also have different patterns of progression within these networks to result in similar clinical symptomatology (Seeley, 2017). We test this hypothesis directly using graph theoretical analyses in our digital dataset.

We are unaware of a previous work using graph theory applied directly to gold-standard postmortem human brain histology data in FTLD or related neurodegenerative disorders. Here, we constructed an adjacency matrix from the Pearson correlation between brain regions sampled to construct a group-level graph for each proteinopathy. Using %AO data for accumulation of pathology, we infer spread of pathology based on the strength of correlation between regions. With this approach we find moderate to high correlation among GM and among WM nodes within both groups, but FTLD-Tau was distinguished by greater correlation between WM-GM node pairs (Figs. 2, 3). This pattern was evident in our subgroup analyses (Fig. 4) and exploratory analysis of hippocampal connectivity (Fig. 5). Interestingly, both groups had similar number of edges (i.e., correlations of disease burden) in the WM graph (Fig. 3), and we hypothesized that WM tau but not WM TDP-43 pathology would associate with a network metric of node integration. To test this hypothesis, we defined nodal strength as the sum of all edges in our proteinopathy-group graphs to test relationships between the severity of pathology at a given node and its participation in the spread of pathology across the network. Our novel findings suggest WM tau burden may contribute to spread of pathology, as it was associated with overall stronger integration of WM nodes in the graph in FTLD-Tau, while in FTLD-TDP this relationship was not apparent, suggesting the correlation observed between nodes with relative low burden of TDP-43 WM burden (Figs. 2, 3) is less likely biologically relevant. Unsurprisingly, node strength was also graphically associated with pathologic burden in GM nodes for both groups, suggesting accumulation of pathology in GM is associated with spread of disease among the graph. Moreover, this relationship appeared stronger in FTLD-TDP compared with FTLD-Tau (Fig. 6) but did not reach statistical significance in the relative sparse sampling of cortical regions (13 total nodes) available in this unique high-density sampling autopsy dataset. Moreover, since we only had a single measurement of pathology at each node per patient, we were limited in our ability to calculate individual-patient level network features to test hypotheses at specific nodes. Thus, we investigated a region-driven hypothesis by using causal mediation analysis to directly test GM-WM relationships between specific brain regions with known anatomic links to progressive disease in FTLD for converging evidence.

Mediation analyses are commonly used in neuroscience to test hypothesized causal chain relationships between imaging or cognitive features (Wager et al., 2008; Poppenk and Moscovitch, 2011). Here, we designated anterior frontotemporal regions (MFC, OFC, STC), generally implicated to be involved in early disease, as predictor variable (X) and the posterior parietal region (ANG) as a dependent variable (Y). By using mean WM pathology %AO as the mediator variable (M) we could test the relative influence of pathology accumulating in WM on the inferred spread of disease from anterior GM regions to the distal parietal lobe GM. As expected, the total pathway of GM in anterior early regions was associated with disease burden in later-affected ANG GM in both groups; however, these associations were uniquely mediated in full or part in each region by WM burden in FTLD-Tau and not FTLD-TDP (Fig. 7). These data align with our graph theoretical approach and suggest that the distinctive high burden of WM pathology in tau may reflect a disease process distinct from FTLD-TDP.

It is interesting to speculate on the cellular mechanisms underlying these findings. Experimental models of tau pathology highlight distinct patterns of cell-to-cell transmission of pathologic forms of tau (Clavaguera et al., 2009). These include strain-like properties of human-brain derived homogenates of various tauopathies injected into mouse brains (Iba et al., 2013; Boluda et al., 2015), often with prominent glial accumulation of tau, particularly across long range WM tracts (Narasimhan et al., 2020). This glial pathology appears in addition to axonal threads, mirroring postmortem findings in human brain (Dickson et al., 2011). In the context of these experimental data, we can hypothesize our current analyses reflect a complex process of tau propagation in the human cortex that may be heavily mediated by glia and WM pathology. Transmission models of TDP-43 are emerging (Nonaka et al., 2013; Feiler et al., 2015; Porta et al., 2018), but thus far suggest a similar cell-to-cell transmission of pathogenic seeds of misfolded TDP-43. In these model systems TDP-43 does accumulate in WM oligodendrocytes but appears to be a later occurring event (Porta et al., 2018). This aligns with postmortem human work, where TDP-43 is most densely accumulated in WM tracts closely associated with GM disease, as exemplified in descending motor pathways in ALS (Braak et al., 2013). Thus, we cannot rule out a contribution of oligodendrocytes in the propagation of TDP-43 pathology in FTLD-TDP, but based on our data here, this may be less influential for cortical spread.

Our analysis is not without limitations. While digital measures of histopathology were carefully performed with an open-source validated approach (Irwin et al., 2016a), there is relatively limited depth of view in 6-µm sections. However, our high-throughput methods facilitated the analysis of over 1,300 datapoints from over 119 autopsy-confirmed FTLD which would not be feasible with traditional stereological methods. Because of the extreme rarity of some subtypes of FTLD, we were unable to examine the full pathologic spectrum of each proteinopathy group. We measured pathologic burden, but other digital metrics of neurodegeneration may inform future models of disease spread as these are developed and validated. Moreover, while our cortical sampling was extensive, we did have missing data for several regions (Table 2) in our older legacy samples. Our statistical methods did account for missing data and the mediation analyses focused on core regions available for most subjects in the study, which provided converging evidence to support our graph theoretical analyses. We focused on cortical regions, but future work can model connectivity with subcortical regions, as digital methods to analyze these complex structures are developed and validated. Finally, we focused on monoproteinopathies in the FTLD spectrum, but our approach can be applied to more complex neurodegenerative disorders with common-co-occurring pathologies, such as AD, to test associations of mixed proteinopathies in aging and dementia (Schneider et al., 2009; Robinson et al., 2018).

Our data support a greater overall influence of WM pathology on the cortical distribution of pathology in tauopathies compared with TDP-43 proteinopathies, which may inform future *in vivo* models of predictive disease and highlight divergent cellular patterns of disease progression between these two clinically-similar but biologically distinct proteinopathies. Moreover, our unique approach opens future opportunities to apply network analyses to gold-standard histopathology data to model microscopic cellular processes in the context of the human brain connectome which can be used to develop and validate *in vivo* imaging metrics sensitive directly to tau and TDP-43 pathology.
